# Chlorhexidine bathing in a tertiary care neonatal intensive care unit: A pilot study

**DOI:** 10.1371/journal.pone.0283132

**Published:** 2023-03-23

**Authors:** Maskit Bar-Meir, Shoshana Bendelac, Irina Shchors

**Affiliations:** 1 Pediatric Infectious Diseases, Shaare-Zedek Medical Center, Jerusalem, Israel; 2 The Faculty of Medicine, Hebrew University, Jerusalem, Israel; 3 Neonatal Intensive Care Unit, Shaare-Zedek Medical Center, Jerusalem, Israel; Guru Gobind Singh Medical College, Faridkot, INDIA

## Abstract

**Background:**

Concerns regarding potential risk of dermal irritation have led to the exclusion of NICU patients from the recommendation regarding the use of 2% chlorhexidine gluconate (CHG) wash for daily skin cleansing to reduce bloodstream infections. Our aim was to assess the safety of 2% CHG bathing in NICU patients.

**Methods:**

The regulator required a stepwise study enrollment to three successive groups: term infants, followed by near-term and pre-term infants. For comparison, we used a cohort of matched controls. A propensity score-adjusted regression model was used to compare the groups.

**Intervention:**

Infants were bathed thrice-weekly with 2% CHG-impregnated washcloths. Participant’s skin was examined daily.

**Results:**

Over a total of 661 days of treatment: 384,129, and 148 days for the term, near-term and pre-term groups, respectively, no skin reactions were observed. The intervention group was generally sicker, however, bloodstream infections were similar between the groups.

**Conclusion:**

For infants >30 weeks and >3 days old, 2% CHG bathing was safe. Large multicenter studies are urgently needed to establish the effectiveness of this practice in the NICU.

## Introduction

Healthcare-associated infections (HAIs) result in significant morbidity and mortality in neonates, including poor neurodevelopmental and growth outcomes in early childhood [1–3], increased hospital stay [1], and increased healthcare costs [4]. Over the past 2 decades, there were slow but steady improvements in these outcomes, with a substantial reduction in HAIs among hospitalized neonates [5]. However, even with the implementation of consensus guidelines and bundled practices [6], infection rates in Israeli neonatal intensive care units (NICUs) remain relatively high [7]. Chlorhexidine gluconate (CHG) is a broad-spectrum topical antiseptic that is used in many different clinical settings to prevent infections [8, 9]. CHG skin cleansing before central venous catheter (CVC) insertion is superior to povidone-iodine in reducing the risk for catheter colonization and catheter- related bloodstream infection (CRBSI) [10, 11]. As a result, the Centers for Disease Control and Prevention (CDC) recommend skin preparation with >0.5% CHG before placement of CVC [12]. Additionally, 2% CHG bathing (skin cleansing) is recommended in adult and pediatric ICUs [13]. A large trial in adults showed that daily bathing with CHG-impregnated washcloths was associated with a 28% lower rate of hospital-acquired bloodstream infections [14]. Nevertheless, the safety data in neonates is limited. Although CHG has been shown to be safe and well tolerated in term neonates exposed to chlorhexidine by different methods, including via vaginal washings, umbilical cord cleansing and whole body cleansing [15], concerns regarding potential risk of dermal irritation or chemical burns have led to the exclusion of NICU patients from the recommendation regarding the use of 2% CHG wash for daily skin cleansing to reduce CRBSI [6]. Whole body bathing with CHG was successfully used in neonates to prevent central line-associated bloodstream infections (CLABSI), as well as for targeted MRSA decolonization [3, 16].

We performed a prospective pilot study to assess the safety and efficacy of skin cleansing with 2% CHG-impregnated washcloths in NICU patients.

## Methods

**Fig 1:**
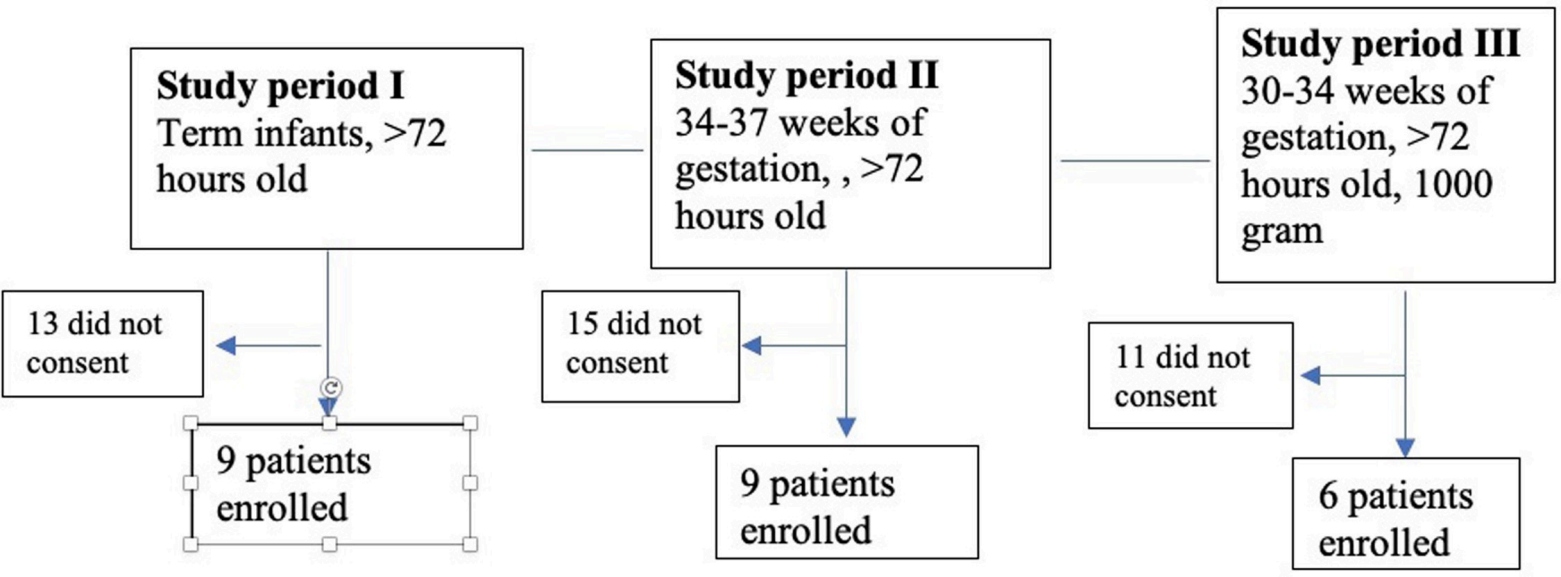
Participant flow diagram.

The NICU of Shaare-Zedek Medical Center (SZMC), an academic tertiary care center, is a 70-bed level III NICU that serves the Jerusalem area. SZMC has >20,000 in-hospital deliveries annually, and the NICU also serves as a referral center for complicated medical or surgical cases from east-Jerusalem NICUs. The NICU has on average 1300 admissions per year, totaling an average of 19,000 annual patient-days. To ensure the safety of the intervention, the Israeli Ministry of Health (MOH) required a stepwise study design of three successive treatment groups as follows: the first group enrolled term infants (gestational age ≥38+0 and >3 days old), the second group with near-term infants (gestational age > 34+0 to 37+6 weeks and > 3 days old) and the third group premature infants (gestational age >30+0 to 33+6,>3 days old and >1000 grams). During each study period, all eligible infants were approached (Fig 1).

Infants, whose parents signed the informed consent, were assigned for bathing three times a week with washcloths impregnated with 2% chlorhexidine gluconate (Clinell ® Chlorhexidine washcloths, GAMA healthcare) for the duration of their NICU stay. One washcloth was wiped over all body surfaces below the jawline and was then allowed to dry naturally without washing with water. Each participant’s skin was examined daily by nursing staff and twice a week by study personnel. Skin reactions were graded and recorded on a standard report form. Grade 3 and 4 skin reactions were defined as serious adverse events (SAEs). Prior to the beginning of the study we have convened all the nurses and reviewed the study details, the possible skin reactions and the grading system. Based on Milstone et al. [17] who reported no serious adverse skin reactions and an incidence of 1.2 minor skin reactions per 1000 patient days, the regulator has approved that if no grade 3 or 4 reactions were encountered after at least 120 patient days per group, enrollment to the subsequent group was allowed. Infants not enrolled in the intervention group continued the regular bathing schedule with water (<32 weeks) or water with mild soap (> = 32 weeks). Skin preparation before CVC insertion as well as cleaning of CVC exit site was performed with 0.5% chlorhexidine gluconate in 70% isopropyl alcohol preparation. This routine remained unchanged throughout the study period.

Although due to the regulatory requirements this was not an experimental design we used a cohort of patients who received the standard bathing for comparison. For each patient in the intervention group, we matched 4 subsequent control patients. Matching was performed for gestational age (±2 weeks) and time of admission (±2 weeks).

Our primary outcome was the safety of 2% CHG washcloths as measured by grade 3 or 4 skin reactions. Skin reactions were classified by grade of severity (grades 1–4 and S1 Fig). Secondary outcomes were number of bloodstream infections, number of new patients colonized with multidrug resistant organism (MDRO- Methicillin-resistant Staphylococcus aureus, Vancomycin-resistant *enterococcus*, Carbapenem-resistant *Acinetobacter baumanii* or Extended-spectrum beta-lactamase producing enterobacteriacea), and the number of antimicrobial therapy courses given for suspected sepsis. During the study period, MDRO universal screening was performed once a week using nasopharyngeal and rectal swabs. Bloodstream infections (BSI) were monitored and reported monthly to the Israeli MOH using the Centers for Disease Control and Prevention (CDC) National Healthcare Safety Network (NHSN) guidelines [18].

The chi-square test (or Fisher-exact test, when appropriate) was used to test the significance of categorical variables. T-test was used to compare continuous variables. Statistical significance was determined using 2-sided p values *(p* < .05).

In order to reduce bias, we used propensity score models with logistic regression for MDRO acquisition as the dependent variable, and linear regression with number of BSI and number of antimicrobial courses as dependent variables. The covariates used for the propensity score included co-morbidities, caesarian section delivery, CVC, mechanical ventilation, and total parenteral nutrition (TPN). All statistical analyses were done using SPSS, version 25.0.

### Ethics

This study was approved by the Israeli Ministry of Health (MOH) and SZMC institutional Helsinky committee. Written informed consent was obtained from the parents of all infants enrolled in the intervention group. For control cases, all data were de-identified, therefore informed consent was waived. This study was registered with ClinicalTrials.gov (Identifier: NCT02537964).

## Results

Overall, 24 infants were enrolled to the intervention group. Of these, 9 were term infants (gestational age ≥38+0), 9 near-term (gestational age > 34+0) and 6 pre-term (gestational age >30+0). The intervention group had a total of 661 days of treatment: 384 for the term group, 129 and 148 days for the near-term and the pre-term groups, respectively. No skin reactions were observed in neither age group throughout the study period.

The demographic and clinical characteristics of the intervention and control groups are presented in Table 1.

**Table 1:** Clinical characteristics and infection-related outcomes of infants undergoing 2% Chlorhexidine gluconate (CHG) bathing versus standard (e.g mild soap/water) bathing.

Characteristic	2% CHG washcloths bathing N = 24	Standard bathing N = 96	*P* value
*Clinical characteristics*			
Mean gestational age, weeks (range)	36.3±3 (30.4–41)	35.6±3 (30–41)	0.3
Mean birth weight, grams ± SD (range)	2691±806 (1530–5000)	2467±667 (1220–4400)	0.1
Male Gender,%	50	54	1
Caesarian section, %	58	34	0.04
Median length of stay, days	18±23	29.5±51	0.05
Congenital Malformations/genetic abnormalities, N (%)	10 (41)	36 (37)	0.4
Central venous catheter, N (%)^1^	18 (75)	31(32)	0.01
Mechanical ventilation, N (%)	13(54)	24(25)	0.006
Total parenteral nutrition	17(70)	36(37)	0.005
*Infection-related outcomes*			
Bloodstream infections, N (%)	7(29)	22(23)	0.2
Multidrug resistant Organisms ^2^, N(%)	4(17)	13 (13.5)	0.3
Mean days to multidrug resistant organism acquisition, (range)	3(0–44)	6.5(0–200)	0.3
Number of antimicrobial therapy courses			
0	3(13)	50(52)	0.02
1	9(37.5)	21(22)	
2	5(21)	8(8)	
3	3(12.5)	9(9)	
4	2(8)	3(3)	
5	2(8)	5(6)	

^1^ CVC included Umbilical artery, Umbilical vein and peripherally inserted central catheters.

^2^ Multidrug resistant organisms include:Methicillin-resistant *Staphylococcus aureus*, Vancomycin-resistant *enterococcus*, Carbapenem-resistant *Acinetobacter baumanii* or Extended-spectrum beta-lactamase producing Enterobacteriaceae.

Since many infants in the term group had surgical pathologies, the intervention group had a higher proportion of CVC use, mechanical ventilation and TPN, compared with the control group. Two patients in the intervention group died. One had multiple congenital anomalies and one had severe hypoxic-ischemic encephalopathy. In both cases, the fatal outcome was judged to be unrelated to the intervention.

BSI and acquisition of MDRO were not significantly different between the 2% CHG and the control group, however antimicrobial use was more frequent in the 2% CHG group (Table 1). This difference in antimicrobial use remained significant in the multivariate analysis: in the propensity score adjusted logistic regression, patients in the control group were less likely to receive antimicrobial therapy (Odds ratio = -0.8, 95%CI:-1.5,-0.2).

## Discussion

CHG bathing implemented for infants >30 weeks of gestational age and >3 days old was safe. Our cohort had a cumulative exposure of 661 days to CHG-bathing and did not present any skin reactions or irritations.

Since this was a pilot study to determine the safety of CHG bathing, it was not powered to detect a potential impact of CHG-bathing on the incidence of BSI or MDRO colonization. Since approximately 40% of the infants in the intervention group were term babies, admitted to the NICU due to either congenital or genetic malformations or due to birth asphyxia, this group was generally sicker, with a higher proportion of CVC use, TPN and mechanical ventilation. The intervention group received significantly more courses of antimicrobial therapy compared with the control group, nevertheless, the incidence of BSI and colonization with MDRO was similar between the groups.

The effectiveness of CHG bathing in reducing CRBSI and BSI in adult and pediatric ICU patients was demonstrated in multiple studies. In a multicenter study performed in pediatric ICUs, it was shown that a child bathed with CHG had a 36% lower risk of bacteremia than a child bathed with standard practices, and that children with CVCs, that were bathed with CHG, had a 34% lower risk of bacteremia [19]. Nevertheless, concerns regarding the safety of CHG in premature infants, especially skin irritation and the potential absorption of CHG through the immature skin, prevented CHG washes from becoming a NICU routine. In real life, however, CHG is frequently used in NICUs. A survey of Canadian and US Children’s hospital showed that 10% of them used CHG for bathing [20]. In a survey from NICUs in England and Wales, over 50% of the units used CHG for CVC skin preparation and maintenance [21].

A study in NICU patients that used a quasi-experimental design to compare CHG bathing in infants with a CVC and a birth weight > 1,000 g, and those with a birth weight <1000 g after day of life 28, before and after the change in policy, found a 65% reduction in CRBSI rates and no serious adverse events [3]. Moreover, CHG skin preparation or the use of CHG-impregnated dressing has been repeatedly shown to be superior to povidone-iodine and 70% alcohol in preventing catheter colonization [22, 23].

One limitation of our pilot study is that CHG blood levels testing was unavailable to us. Although it was shown that some percutaneous absorption of CHG occurs at trace levels, particularly in very young preterm infants [15], there have been no reports of adverse consequences as a result of this absorption, and no data to suggest that CHG trace levels have clinical importance [9]. The monitoring of possible adverse skin reactions was not performed by fixed persons, but rather by the nurses that took care of the babies and by the study personnel, therefore, inter or intra-observer variability may be a concern. However, grades 2–4 skin reactions are unlikely to be under-diagnosed.

Another major limitation is that due to concerns regarding the safety of 2% CHG bathing, we were required by the regulator to proceed gradually from term infants to near-term and pre-term, with an interim analysis to ensure no serious adverse events occur. Only approximately 40% of parents offered to enroll their infants in the study, indeed signed the informed consent. Luckily, With the implementation of bundled practices, the incidence of BSI in the NICU has reduced substantially. However, in order to design a randomized study with enough statistical power to detect a potential reduction in infection-related outcomes (e.g BSI or MDRO acquisition), many hundreds of patients will have to be enrolled.

A delicate balance must be maintained between the inherent rigorous regulatory oversight in any research and the urgent need to make effective and evidence-based tools, such as CHG-bathing, available to high-risk populations, such as neonates and pre-term newborns.

In conclusion, the use of 2% CHG cloths for bathing infants >30 weeks and >3 days old was safe. Large multicenter studies are urgently needed to establish the effectiveness of this practice in the NICU.

## Supporting information

S1 Fig Classification of skin reactions by grade of severity. (DOCX)

S1 File Study protocol. (DOCX)

S2 File TREND checklist. (DOC)

S3 File Minimal underlying data set. (CSV)
