# Supplementary material for: Chlorhexidine bathing in a tertiary care neonatal intensive care unit: A pilot study
Source: PLoS One. 2023 Mar 23;18(3):e0283132. doi: 10.1371/journal.pone.0283132 (PMC10035923; doi:10.1371/journal.pone.0283132)
Supplement: S1 Fig — (DOCX) [file pone.0283132.s001.docx]

**Figure S1. Classification of skin reactions by grade of severity**
