# Supplementary material for: Chlorhexidine bathing in a tertiary care neonatal intensive care unit: A pilot study
Source: PLoS One. 2023 Mar 23;18(3):e0283132. doi: 10.1371/journal.pone.0283132 (PMC10035923; doi:10.1371/journal.pone.0283132)
Supplement: S1 File — (DOCX) [file pone.0283132.s002.docx]

STUDY PROTOCOL-ammended, June 15, 2016

Evaluation of the Effectiveness of Chlorhexidine Bathing in Reducing Nosocomial Infections in the Neonatal Intensive Care Unit

Principal Investigator:

Maskit Bar-Meir, M.D.

Pediatric Infectious Diseases,

Shaare-Zedek Medical Center, Jerusalem, Israel

Study site: Neonatal Intensive care unit , Shaare-Zedek Medical Center

Version 03

June 15,2016

**1. Study Synopsis**

| Protocol Title | Evaluation of the Effectiveness of Chlorhexidine Bathing in Reducing Nosocomial Infections in the Neonatal Intensive Care Unit |
| --- | --- |
| Protocol Number |  |
| Sponsor |  |
| Primary objective | To determine if daily bathing with chlorhexidine impregnated  washcloths will reduce the incidence of bloodstream infections within the neonatal intensive care unit (NICU) . |
| Study design | This is a prospective, controlled; crossover study of daily bathing with no-rinse, 2% CHG impregnated washcloths versus bathing with water/soap or water according to gestational age and weight (e.g. standard bathing). The trial will take place in the NICU.  **In the preliminary phase of the study we will establish the safety of chlorhexidine bathing using Clinell ® Chlorhexidine wash cloths on three groups of patients: term infants admitted to the NICU; late preterm infants (34-37 weeks); preterm infants 30-34 weeks of gestation. Interim analysis for adverse events will be performed after each group of patients.**  **In the subsequent phases of the study,** all infants admitted to the NICU and enrolled in the study will be bathed three times a week with Clinell ® Chlorhexidine wash cloths during the initial 6-months study period (intervention), followed by standard bathing during the second 6-months period, then again intervention period for 6 months and standard bathing for 6-months. Total study period- 2 years.  Data collection will include all bloodstream infections as well as surveillance cultures |
| Number of subjects | Approximately 800 patients in both arms  **Up to 60 patients (at least 200 patient days) in the preliminary study** |
| Study population | Infants **>=10** days ,weighing **>=1500** grams admitted to the NICU |
| Duration of participation | Duration of NICU stay OR for the duration of invasive hardware (e.g intravacular catheters, nasogastric tubes etc. ) |
| Treatment | Bathing three times a week with either:  a.2% chlorhexidine gluconate cloth  b. water/mild soap +water |
| Assessment of efficacy | The primary efficacy endpoint will be the reduction in bloodstream infections incidence during those study periods where the 2% Chlorhexidine Gluconate Cloth will be utilized.  Additional study endpoints include overall incidence of colonization with multi-drug resistant organisms (MDRO) and time to colonization |
| Safety | Safety will be assessed through the monitoring of adverse events  associated with bathing products to include any skin rashes or  hypersensitivity reactions. |

**2. Study personnel**

Principal Investigator:

Maskit Bar-Meir, M.D.

Pediatric Infectious Diseases,

Shaare-Zedek Medical Center, Jerusalem, Israel

Office: +947-2-6666340

Fax: +947-2-6666840

Mail: mbarmeir@gmail.com

Research coordinator:

**TBD**

3. Background

Preterm delivery rates in developed countries, including Israel, vary between 5-11%^[[1]](#endnote-1),^^[[2]](#endnote-2)^. Nosocomial infections (NIs) are a major health problem among premature infants, and are associated with increased morbidity, mortality, length of hospital stay, and costs^[[3]](#endnote-3),^^[[4]](#endnote-4)^. Overall infection rates range from 8.9 to 62 infections per 1,000 patient days^[[5]](#endnote-5)^’^[[6]](#endnote-6)^. Over the last 25 years, the improvement in survival of premature infants was paralleled by an increased rate of nosocomial infections (NI), especially among very low birth weight (VLBW) infants, who develop NI in 21-43% of cases^3^. This incidence is inversely related to gestational age and birth weight and directly correlated with severity of illness, length of stay, ventilation, central catheters, parenteral nutrition days, and broad spectrum antibiotics.

The emergence of multidrug- resistant organisms (MDRO) in acute-care settings, including the neonatal intensive care unit (NICU) , complicates the antimicrobial therapy and highlights the urgent need to limit the spread of such organisms and reduce the rate of NIs. Resistant organisms such as methicillin-resistant *Staphylococcus aureus* (MRSA), extended-spectrum beta-lactamase producing enterobacteriacae (ESBL) and carbapenem resistant *Acinetobacter baumanii* (CRAB) have caused outbreaks with associated morbidity and mortality in NICUs around the world ^[[7]](#endnote-7)^’^[[8]](#endnote-8)^’^[[9]](#endnote-9)^ . A stay in the NICU for > 1 week is a prominent predictor of colonization with resistant organisms^[[10]](#endnote-10)^. Strategies that have been utilized to limit the spread of MDRO within ICU’s have included stricter attention to barrier precautions following identification of patients colonized with these organisms, as well as improved handwashing. One prominent strategy that has emerged as recommended by recent SHEA guidelines ^[[11]](#endnote-11)^suggests that hospitals should adopt more aggressive active surveillance culturing to identify patients colonized with MDRO at the time of admission and periodically during their hospital stay. It is advocated, that under this “search and isolate” strategy, once reservoirs of resistant organisms within the hospital are identified, nosocomial transmission can be effectively eliminated through the use of strict barrier precautions and hand hygiene for these previously unidentified patients. However, this “search and isolate” strategy has multiple flaws. First, it is costly and requires high level of compliance with contact precautions and hand hygiene among healthcare personnel. Second, this strategy does little to eradicate colonization.

*As long as patients remain colonized, the opportunity for transmission exists. Therefore, a strategy that may address skin carriage of pathogens has the potential to reduce horizontal transfer to other patients as well as reduce the colonized patient’s risk to develop infection.*

The guidelines for preventing intravascular catheter-related infections published by the Centers for Disease Control and Prevention (CDC) recommend daily skin cleansing of ICU patients with 2% chlorhexidine to reduce catheter-related bloodstream infections (BSI)^[[12]](#endnote-12)^, but could not recommend its use in infants < 2 months of age due to lack of safety and efficacy data.

Chlorhexidine gluconate (CHG) is a broad spectrum antiseptic agent that maintains residual activity on patient’s skin. As a result, it may reduce efficiently the bacterial burden on the skin and prevent secondary environmental contamination ^[[13]](#endnote-13)^.It is now recognized that chlorhexidine is superior to other agents in site preparation for invasive procedures. The use of chlorhexidine reduces residual skin organisms as well as inhibits their rebound growth and has been demonstrated to reduce central-line associated bloodstream infection (CLABSI) in comparison to other skin disinfectant products such as povidone-iodine^[[14]](#endnote-14)^.

The same properties that make chlorhexidine an effective agent in the prevention of CLABSI have been utilized in selective settings to reduce the incidence of MRSA within the ICU.

Chlorhexidene has been used successfully to eradicate MRSA skin colonization ^[[15]](#endnote-15)^’^[[16]](#endnote-16)^.

The reduction in skin colonization with MRSA is thought to lead to reduced risk for horizontal transmission of MRSA within the ICU environment.

The use of chlorhexidine has been used successfully in the control of a number of MRSA

outbreaks within the NICU setting ^[[17]](#endnote-17)^’^[[18]](#endnote-18)^’^[[19]](#endnote-19)^. Although the selective use of chlorhexidine for catheter site preparation and in the selective treatment of MRSA colonized patients

during outbreaks has been investigated , there is little study of the potential utility of

more wide scale use of chlorhexidine in daily bathing routines , especially in the NICU. With

daily chlorhexidine bathing , there is the potential to reduce a number of NI, including CLABSI by reducing bacterial burdens on the skin. Reductions in resident bacteria on the skin could lead to reduced horizontal transmission of multiresistant bacterial pathogens and better outcomes following invasive procedures .

*Daily bathing to produce a state of skin asepsis is an attractive theorectical means to reduce nosocomial infections because it represents a simple intervention that could be applied universally with relatively little effort.*

A recent multicenter study examined the use of chlorhexidine-impregnated washcloths among adult patients at nine intensive care and bone marrow transplantation units. The chlorhexidine group had 23% lower rate of MDRO acquisition and a 28% lower rate of BSI compared with the control group ^13^. A systematic review of 15 studies, 9 of which were randomized controlled trials, provided support for the usefulness of CHG in a variety of healthcare settings including intensive care units, internal and surgical hospital wards ^[[20]](#endnote-20)^ .

Studies that examined the use of CHG in neonates were conducted mainly in developing countries and evaluated single whole body cleansing of the newborn immediately after birth, with or without maternal vaginal cleansing. CHG concentrations varied between 0.25% and 1%, and its use was associated with a reduction in neonatal mortality in high-mortality settings, but did not show benefit in low neonatal mortality settings^[[21]](#endnote-21)^’^[[22]](#endnote-22)^’^[[23]](#endnote-23)^’^[[24]](#endnote-24)^’^[[25]](#endnote-25)^’^[[26]](#endnote-26)^ . A large multicenter study in 10 pediatric intensive care units (PICUs) in the USA found that the incidence of bacteremia was 36% lower among patients receiving daily CHG bathing (3.28 per 1,000 days, 95%CI 2.27–4.58)) compared with patients receiving standard bathing practices (4.93 per 1,000 days, 95%CI 3.91–6.15 ). There were no serious study related adverse events^[[27]](#endnote-27)^.

Despite the fact that CHG was not approved by the US Food and Drug Administration for use in infants <2 months of age, it was used in large well-designed clinical trials on tens of thousands of neonates without reported serious adverse events^[[28]](#endnote-28)^’^[[29]](#endnote-29)^. Moreover, a recent survey in US NICUs found that the majority were using CHG, mainly for central venous catheter (CVC) site preparation and CVC maintenance ^[[30]](#endnote-30)^.

A recent study examined the effect of 2%CHG bathing on prevention of central-line associated bloodstream infection (CLABSI) in the NICU. Eligible infants were those with CVC weighing >1000 grams or those weighing <1000 grams after day 28 of life. Using a quasi-experimental study design the authors compared the rate of infection among infants before and after the intervention and demonstrated a decrease from 6 to 1.92/1000 CVC-days. No adverse events were reported^[[31]](#endnote-31)^.

Recently, the Israeli Ministry of health published guidelines that endorsed the use of 2%CHG solutions for the insertion of umbilical catheters. Shortly after, a national program for the reduction of NIs in Israeli NICUs was launched and encouraged the use of bundled practices including the use of 2%CHG for insertion and maintenance of central and peripheral catheters. The rate of CLABSI and BSI remains high in the NICU despite compliance with consensus guidelines and infection control measures. Lack of evidence from randomized trials prompt us to examine the usefulness of bathing with CHG to reduce the risk of CLABSI and BSI among infants in the NICU.

**4. Expected significance**

National data from the USA indicate that almost a quarter of very low birth weight infants will have one or more episodes of sepsis. Compared with infants without sepsis, infants with sepsis had a significantly longer hospital stay (79 vs.60 days), had a 2-fold increase in the number of days on mechanical ventilation, and had a significantly higher mortality rate (18% vs 7%)3. Another American study estimated that, on average, the cost of NICU care was $16,800 greater per infant who experienced NICU-associated BSI^[[32]](#endnote-32)^. Approximately 7% of infants, or >12,000 infants, are born prematurely in Israel every year^[[33]](#endnote-33)^. Extrapolating the American data to the Israeli NICU translates into a major health problem. The Ministry of Health and the Israel Neonatal Society have recently launched a national program aimed at minimizing the rate of NI in the Israeli NICUs^33^. In our NICU we have adopted this program and have increased staff education, active monitoring of compliance with guidelines, as well as adoption of the “search and isolate” policy, with twice a week screening of patients for colonization with MDRO.

Despite these measures, BSI rates remain high in our NICU, which prompt us to search for additional interventions such as CHG baths. The goal of the currently proposed study is to determine if universal use of a chlorhexidine based bathing system for patients in the NICU will decrease skin bacterial burden and lead to decreased horizontal transmission of MDRO.

Additionally, we hypothesize that reduced skin colonization with opportunistic bacterial

pathogens will result in a reduction in the rate of CLABSI, BSI and clinical sepsis in comparison to regular bathing procedures.

**5. Research design and Methods**

**5.1 Hypothesis:**

A change in the regular bathing procedures in the NICU to utilize products containing chlorhexidine will result in a reduction in the number of colonizing bacteria on the

skin of patients. Reduced colonization of the skin (skin asepsis) will lower the incidence of

nosocomial transmission of bacteria in the ward and decrease incident cases of new

bacteremias .

**5.2 Design**

This is a prospective, controlled; crossover study of daily bathing with no-rinse, 2% CHG impregnated washcloths versus bathing with water/soap or water according to gestational age and weight (e.g. standard bathing).

The primary study outcome is CLABSI/ BSI and clinical (culture-negative) sepsis.

Secondary study outcomes are rate and time to colonization with pathogenic bacteria.

**5.3 Study Setting**

The study will take place in the NICU of Shaare-Zedek Medical Center

**5.4 Preliminary study**

**In order to establish safety , we will enroll infants admitted to the NICU to three successive treatment groups , as follows: the first group will include term infants admitted to the NICU (gestational age ≥37+6 and >7 days old), the second group will include near-term infants (gestational age > 34+0 to 36+6 weeks and >7 days old) and the third group will include premature infants (gestational age >30+0 to 33+6 and >=10 days old). We intend to enroll up to 20 patients in each group, for a minimum of 200 patient days per group (see sample size estimation). All infants will be assigned for bathing three times a week with washcloths impregnated with 2% chlorhexidine gluconate (Clinell ® Chlorhexidine wash cloths, GAMA healthcare) for the duration of their NICU stay. The infant’s skin will be monitored continuously as described below (see ‘6.0 Safety and monitoring’ and appendix 2). In case of grade 3 or 4 reactions, the study interventions will be temporarily suspended, and the adverse event will be reported to the Helsinky committee and to the study steering committee. If no grade 3 or 4 reactions will be encountered after at least 200 patient days per group, enrollment to the subsequent group will be performed (e.g if no grade 3-4 reaction will be encountered for 200 patient days in group 1, we will start enrolling patients for group 2 etc.). If no grade 3-4 dermatitis is encountered in the preliminary study, we will initiate enrollment as described below (‘5.5 intervention’)**

**5.5 Intervention**

All infants in the NICU eligible for the study will be assigned for bathing three times a week with washcloths impregnated with 2% chlorhexidine gluconate (Clinell ® Chlorhexidine wash cloths, GAMA healthcare) during the initial 6-months study period (intervention), followed by standard bathing during the second 6-months period, then again intervention period for 6 months and standard bathing for 6-months. Total study period- 2 years.

**Inclusion criteria**: infants weighing ≥1500 grams and ≥10 days of age; parents’ informed consent

**Exclusion criteria:** infants weighing <1500 grams or <10 days of age**;** parents’ refusal to sign informed consent.

For standard bathing procedure see appendix 1.

Before the study initiation, as well as at the beginning of each study period, nurses will be instructed on the proper technique for bathing patients. Bathing will be performed according to product brochure, three times a week.

**5.6 Outcome measures**

A. Number of BSI/CLABSI/clinical sepsis events per 1000 patient days during the intervention periods compared with the control periods.

B. Number of new patients colonized with MRSA, VRE, CRAB or ESBL per total number of patients, monthly range and variance.

C. Number of new patients colonized with MRSA, VRE, CRAB or ESBL per 1000 eligible patient days [ Total patient days – total patient days for patients identified with MDRO]

D. Time to colonization with MDRO during the intervention periods compared with the control periods.

**5.7 Data collection**

Each patient admitted to the NICU during the study periods and meeting the inclusion criteria, will be recorded and assigned a specific study number. The dates of admission and dates of discharge will be recorded and used to calculate length of stay and to determine the

incidence of nosocomial infections based on microbiological data. Clinical and laboratory data, including the use of invasive device (intravascular devices, mechanical ventilation, feeding tubes) will be collected on standardized forms. The data will be coded under a password protected database and linked only to study patient identifier.

Infections and MDRO acquisitions will be monitored during the study period. Events occurring within 2 days after the transition will be assigned to the previous bathing period.

For infection surveillance, daily review of cultures and new orders for antibiotic therapy will be performed for all study participants. Infectious events will be evaluated by an independent investigator blinded to the intervention period.

Active surveillance testing for MDRO will be performed once every two weeks during the study period. Swabs from the nasopharynx (for MRSA)] and from the rectal area (for ESBL producing organisms, VRE and CRAB) will be obtained by unit staff and processed in the microbiology laboratory of Shaare-Zedek Medical Center.

**5.8 Trial oversight:**

The steering committee of the study will include, except for the PI, two other Infectious Disease physicians (Dr Wiener-Well and Dr. Meged). There will be biweekly discussions of the progress

report, reporting of compliance with active surveillance, reporting of compliance with

bathing, and any adverse reactions or other problems.

**5.9 Statistical analyses**

Changes in the rates of BSI and clinical sepsis will be compared between the intervention and the control period. Continuous variables will be examined with the use of two-sample t-tests and linear regression modeling, and categorical variables will be examined by means of Fisher’s exact test. Cox proportional-hazards regression model will be used to compare the time from admission until the first primary bloodstream infection , clinical sepsis or acquisition of MDRO between the control and intervention periods.

**Sample size calculation:** based on our ongoing surveillance, there are 2-3 BSI events per 1000 patients –days in our NICU. In order to detect a 50% reduction in BSI rates , we will need 37,096 patient days (18,548 in each group) in order to have 80% power to reject the null hypothesis, with 0.05 level of significance. Currently, our average annual census is 45,000 patient days. therefore, a 2-year study will have >80% power to reject the null hypothesis (the BSI rates in the control period equal the rates in the intervention period).

**For the preliminary study, we used the data in Milstone et al. ^18^ who reported no serious adverse skin reactions and an incidence of 1.2 minor skin reactions per 1000 patient days. A sample of 500 patient days will enable us to detect a ten-fold higher rate of minor skin reactions. Therefore at least 200 patient days in each group of the preliminary study (>600 patient days), would suffice.**

**6.0 Safety and monitoring.**

A study that evaluated the tolerability of 2% CHG for catheter insertion antisepsis among infants ≥1500 grams and ≥7 days old found no significant dermatitis reactions^[[34]](#endnote-34)^. Seven of 48 infants had measurable CHG levels in the blood. In a trial that evaluated a multifactorial approach to reduce the rates of CLABSI, Andersen et al. noted that 4 of 36 neonates <1000g developed contact dermatitis after a 2% CHG scrub. There were no episodes of contact dermatitis in 49 study participants who were ≥1000g^[[35]](#endnote-35)^. The alternative for antisepsis in neonates is povidone-iodine. However, iodine absorption that may compromise thyroid function is one reason to explore the use of CHG. Although CHG was reported to be absorbed through neonatal skin in several trials, no significant systemic side effects were reported^[[36]](#endnote-36)^’^[[37]](#endnote-37)^. Animal trials showed no significant toxicity from CHG^[[38]](#endnote-38)^. The US Food and Drug Administration (FDA) states on the drug facts box of 2% CHG cloth:” use with care in premature infants or infants under 2 months of age. These products may cause irritation or chemical burns”.

Each participant’s skin will be examined daily by nursing staff and twice a week by study personnel.

Skin reactions will be graded and recorded on a standard report form (appendix 2).

Grade 3 and 4 skin reactions will be reported as serious adverse events.

**6.1 Human subject research considerations**

*6.1.1 Human subjects involvement and characteristics*

This is a crossover study. The control will be the current bathing procedures in our NICU. The “intervention” will be the use of 2% chlorhexidine gluconate cloth which is FDA

approved for adult patient perioperative skin preparation and for use to reduce bacteria that can

potentially cause skin infection. ^[[39]](#endnote-39)^. The FDA states on the drug facts box of 2% CHG cloth:” use with care in premature infants or infants under 2 months of age. These products may cause irritation or chemical burns”.

Monitoring of any unexpected adverse effects or serious adverse events related to study

procedures will be the responsibility of the principal investigator and will be reported

immediately to the steering committee as well as the IRB. (see appendix c for SAE report form)

For the purposes of this research protocol *unexpected adverse events* and *serious adverse*

*events* will be defined as follows:

*Unexpected adverse event:* Any adverse reaction or experience that is not listed in the

current labeling for the drug product or investigators brochure. Known adverse reactions

to chlorhexidene containing topical products include irritation, sensitization, and generalized

allergic reactions. Chlorhexidine should be kept out of the ears and eyes.

*Serious adverse drug event.* Any adverse reaction or experience occurring at any dose that

results in any of the following outcomes: Death, a life-threatening adverse reaction,

inpatient hospitalization or prolongation of existing hospitalization and a persistent or significant

disability/incapacity.

*6.1.2 Potential risks*

Potential recognized risks of bathing with chlorhexidine include local skin irritation, allergic reaction and irritation of mucous membranes

6.1.3 Potential Benefit

The purpose of this study is to demonstrate that the routine use of chlorhexidine in bathing

practices will lead to reduced skin colonization with pathogens and reduced nosocomial bacteremias. Such reductions could have profound effects on associated morbidity and mortality. Since the intervention that is proposed is of minimal risk to the involved patients the potential benefits far outweigh any potential risk to individual patients.

*6.1.4* Importance of the knowledge to be gained

If changes in bathing practices prove to be effective in reducing Nosocomial infections, this

would represent a simple intervention that could be applied broadly to all NICUs

Appendix 1.

Standard Bathing procedures

| **Gestational week** | **Age at Onset of Bathing** | **Frequency** | **Product** |
| --- | --- | --- | --- |
| <26 | 3 weeks | Once a week | Water |
| 27-30 | 2 weeks | Once a week | Water |
| 31-32 | 1 week | Three times a week | Water |
| >=33 | 1 week | Three times a week | Water+mild soap |
| Term | Day 2 | Three times a week | Water+mild soap |

Appendix 2.

7. References

1. Goldenberg RL, Culhane JF, Iams JD, Romero R. Epidemiology and causes of preterm birth. Lancet. 2008 Jan 5;371(9606):75-84. [↑](#endnote-ref-1)
2. <http://www.cdc.gov/reproductivehealth/maternalinfanthealth/PretermBirth.htm> [↑](#endnote-ref-2)
3. Stoll BJ, Hansen N, Fanaroff AA, Wright LL, Carlo WA, Ehrenkranz RA, Lemons JA et al. Late-onset sepsis in very low birth weight neonates: the experience of the NICHD Neonatal Research Network. Pediatrics. 2002 Aug;110(2 Pt 1):285-91. [↑](#endnote-ref-3)
4. Mahieu LM, Buitenweg N, Beutels P, De Dooy JJ. Additional hospital stay and charges due to hospital- acquired infections in a neonatal intensive care unit. J Hosp Infect. 2001 Mar;47(3):223-9. [↑](#endnote-ref-4)
5. Sohn AH, Garrett DO, Sinkowitz-Cochran RL, et al. Prevalence of nosocomial infections in neonatal intensive care unit patients: Results from the first national point-prevalence survey. J Pediatr 2001;139:821-827. [↑](#endnote-ref-5)
6. Nagata E, Brito AS, Matsuo TL. Nosocomial infections in a neonatal intensive care unit: Incidence and risk factors. Am J Infect Control 2002;30:26-31. [↑](#endnote-ref-6)
7. Cipolla D, Giuffrè M, Mammina C, Corsello G. Prevention of Nosocomial infections and surveillance of emerging resistances in NICU. J Matern Fetal Neonatal Med. 2011 Oct;24

   Suppl 1:23-6. [↑](#endnote-ref-7)
8. Giuffrè M, Cipolla D, Bonura C, Geraci DM, Aleo A, Di Noto S et al. Epidemic spread of ST1-MRSA-IVa in a neonatal intensive care unit, Italy. BMC Pediatr. 2012 Jun 8;12:64. [↑](#endnote-ref-8)
9. Cantey JB, Sreeramoju P, Jaleel M, Treviño S, Gander R, Hynan LS et al. Prompt control of an outbreak caused by extended-spectrum β-lactamase-producing Klebsiella pneumoniae in a neonatal intensive care unit. J Pediatr. 2013 Sep;163(3):672-9.e1-3. [↑](#endnote-ref-9)
10. Navarro LR, Pekelharing-Berghuis M, de Waal WJ, Thijsen SF. Bacterial colonization patterns in neonates transferred from neonatal intensive care units. Int J Hyg Environ Health. 2011 Mar;214(2):167-71. [↑](#endnote-ref-10)
11. Muto CA, Jernigan JA, Ostrowsky BE, Richet HM, Jarvis WR, Boyce JM et al. SHEA guideline for preventing nosocomial transmission of multidrug-resistant strains of Staphylococcus aureus and enterococcus. Infect Control Hosp Epidemiol. 2003 May;24(5):

    362-86. [↑](#endnote-ref-11)
12. O'Grady NP, Alexander M, Burns LA et al. Healthcare Infection Control Practices Advisory Committee (HICPAC) Summary of recommendations: Guidelines for the Prevention of Intravascular Catheter- related Infections. Clin Infect Dis. 2011 May;52(9):1087-99. [↑](#endnote-ref-12)
13. Climo MW, Yokoe DS, Warren DK et al. Effect of daily chlorhexidine bathing on hospital-acquired infection. N Engl J Med. 2013 Feb 7;368(6):533-42. [↑](#endnote-ref-13)
14. Chaiyakunapruk N, Veenstra DL, Lipsky BA, Sullivan SD, Saint S. Vascular catheter site care: the clinical and economic benefits of chlorhexidine gluconate compared with povidone iodine. Clin Infect Dis. 2003 Sep 15;37(6):764-71. [↑](#endnote-ref-14)
15. Sandri AM, Dalarosa MG, Ruschel de Alcantara L, da Silva Elias L, Zavascki AP.

    Reduction in incidence of nosocomial methicillin-resistant Staphylococcus aureus

    (MRSA) infection in an intensive care unit: role of treatment with mupirocin

    ointment and chlorhexidine baths for nasal carriers of MRSA. Infect Control Hosp

    Epidemiol. 2006 Feb;27(2):185-7. [↑](#endnote-ref-15)
16. Girou E, Pujade G, Legrand P, Cizeau F, Brun-Buisson C. Selective screening of carriers for control of methicillin-resistant Staphylococcus aureus (MRSA) in high-risk hospital areas with a high level of endemic MRSA. Clin Infect Dis. 1998 Sep;27(3):543-50. [↑](#endnote-ref-16)
17. Khoury J, Jones M, Grim A, Dunne WM Jr, Fraser V. Eradication of methicillin-resistant Staphylococcus aureus from a neonatal intensive care unit by active surveillance and aggressive infection control measures. Infect Control Hosp Epidemiol. 2005 Jul;26(7):616-21. [↑](#endnote-ref-17)
18. Milstone AM, Song X, Coffin S, Elward A; Society for Healthcare Epidemiology of America's Pediatric Special Interest Group. Identification and eradication of methicillin-resistant Staphylococcus aureus colonization in the neonatal intensive care unit: results of a national survey. Infect Control Hosp Epidemiol. 2010 Jul;31(7):766-8. [↑](#endnote-ref-18)
19. Regev-Yochay G, Rubinstein E, Barzilai A et al. Methicillin-resistant Staphylococcus aureus in neonatal intensive care unit. Emerg Infect Dis. 2005 Mar;11(3):453-6. [↑](#endnote-ref-19)
20. Afonso E, Llauradó M, Gallart E. The value of chlorhexidine gluconate wipes and prepacked washcloths to prevent the spread of pathogens--a systematic review. Aust Crit Care. 2013 Nov;26(4):158-66. [↑](#endnote-ref-20)
21. Taha TE, Biggar RJ, Broadhead RL, et al. Effect of cleansing the birth canal with

    antiseptic solution on maternal and newborn morbidity and mortality in Malawi: clinical

    trial. *BMJ*. 1997;315:216–219. [↑](#endnote-ref-21)
22. Cutland CL, Madhi SA, Zell ER, et al.; PoPS Trial Team. Chlorhexidine maternal-vaginal

    and neonate body wipes in sepsis and vertical transmission of pathogenic bacteria in

    South Africa: a randomised, controlled trial. *Lancet*. 2009;374:1909–1916. [↑](#endnote-ref-22)
23. McClure EM, Goldenberg RL, Brandes N, et al.; CHX Working Group. The use

    of chlorhexidine to reduce maternal and neonatal mortality and morbidity in low-

    resource settings. *Int J Gynaecol Obstet*. 2007;97:89–94. [↑](#endnote-ref-23)
24. Mullany LC, Darmstadt GL, Tielsch JM. Safety and impact of chlorhexidine

    antisepsis interventions for improving neonatal health in developing countries. *Pediatr*

    *Infect Dis J*. 2006;25:665–675. [↑](#endnote-ref-24)
25. Blencowe H, Cousens S, Mullany LC, et al. Clean birth and postnatal care practices to

    reduce neonatal deaths from sepsis and tetanus: a systematic review and Delphi estimation

    of mortality effect. *BMC Public Health*. 2011;11(suppl 3):S11. [↑](#endnote-ref-25)
26. Sankar MJ, Paul VK. Efficacy and safety of whole body skin cleansing with chlorhexidine

    in neonates--a systemic review. Pediatr Infect Dis J. 2013 Jun;32(6):e227-34. [↑](#endnote-ref-26)
27. Milstone AM, Elward A, Song X et al. Pediatric SCRUB Trial Study Group. Daily chlorhexidine bathing to reduce bacteraemia in critically ill children: a multicentre, cluster-randomised, crossover trial. Lancet. 2013 Mar 30;381(9872):1099-106. [↑](#endnote-ref-27)
28. Mullany LC, Darmstadt GL, Khatry SK, et al. Topical applications of chlorhexidine to

    the umbilical cord for prevention of omphalitis and neonatal mortality in southern Nepal:

    a community-based, cluster-randomised trial. *Lancet* 2006;367:910–918. [↑](#endnote-ref-28)
29. Tielsch JM, Darmstadt GL, Mullany LC, et al. Impact of newborn skin cleansing

    with chlorhexidine on neonatal mortality in southern Nepal: a community-based,

    cluster-randomized trial. *Pediatrics* 2007;119(2): e330–e340. [↑](#endnote-ref-29)
30. Tamma PD, Aucott SW, Milstone AM. Chlorhexidine use in the neonatal intensive care unit: results from a national survey. Infect Control Hosp Epidemiol. 2010 Aug;31(8):846-9. [↑](#endnote-ref-30)
31. Quach C, Milstone AM, Perpête C, Bonenfant M, Moore DL, Perreault T. Chlorhexidine bathing in a tertiary care neonatal intensive care unit: impact on central line-associated bloodstream infections. Infect Control Hosp Epidemiol. 2014 Feb;35(2):158-63. [↑](#endnote-ref-31)
32. Donovan EF, Sparling K, Lake MR, Narendran V, Schibler K, Haberman B, Rose B, Meinzen-Derr J; Ohio Perinatal Quality Collaborative. The investment case for preventing NICU associated infections. Am J Perinatol. 2013 Mar;30(3):179-84. [↑](#endnote-ref-32)
33. <http://www.health.gov.il/NewsAndEvents/SpokemanMesseges/Pages/17112014_1.aspx> [↑](#endnote-ref-33)
34. Garland JS, Alex CP, Uhing MR, Peterside IE, Rentz A, Harris MC. Pilot trial to compare tolerance of chlorhexidine gluconate to povidone-iodine antisepsis for central venous catheter placement in neonates. J Perinatol. 2009 Dec;29(12):808-13. [↑](#endnote-ref-34)
35. Andersen C, Hart J, Vemgal P, Harrison C. Prospective evaluation of a multi-factorial prevention strategy on the impact of nosocomial infection in very-low-birthweight infants. J Hosp Infect. 2005 Oct;61(2):162-7. [↑](#endnote-ref-35)
36. Garland JS, Buck RK, Maloney Pet al. Comparison of 10% povidone-iodine and 0.5% chlorhexidine gluconate for the prevention of peripheral intravenous catheter colonization in neonates: a prospective trial. Pediatr Infect Dis J. 1995 Jun;14(6):510-6. [↑](#endnote-ref-36)
37. Aggett PJ, Cooper LV, Ellis SH, McAinsh J. Percutaneous absorption of chlorhexidine in neonatal cord care. Arch Dis Child. 1981 Nov;56(11):878-80. [↑](#endnote-ref-37)
38. Case DE. Safety of Hibitane. I. Laboratory experiments. J Clin Periodontol. 1977 Dec;4(5):66-,2. [↑](#endnote-ref-38)
39. Case DE. Safety of Hibitane. I. Laboratory experiments. J Clin Periodontol. 1977 Dec;4(5):66-,2. [↑](#endnote-ref-39)
